# Assessing the Risk Factors For Diagnosed Symptomatic Dry Eye Using a Smartphone App: Cross-sectional Study

**DOI:** 10.2196/31011

**Published:** 2022-06-22

**Authors:** Ngamjit Kasetsuwan, Olan Suwan-Apichon, Kaevalin Lekhanont, Varintorn Chuckpaiwong, Usanee Reinprayoon, Somporn Chantra, Vilavun Puangsricharern, Lalida Pariyakanok, Pinnita Prabhasawat, Nattaporn Tesavibul, Winai Chaidaroon, Napaporn Tananuvat, Chakree Hirunpat, Nauljira Prakairungthong, Wiwan Sansanayudh, Chareenun Chirapapaisan, Pakornkit Phrueksaudomchai

**Affiliations:** 1 Department of Ophthalmology Faculty of Medicine Chulalongkorn University Bangkok Thailand; 2 Cornea and Refractive Surgery Society of Thailand Department of Ophthalmology King Chulalongkorn Memorial Hospital Bangkok Thailand; 3 Excellence Center of Cornea and Limbal Stem Cell Transplantation Faculty of Medicine, Chulalongkorn University Bangkok Thailand; 4 Department of Ophthalmology Faculty of Medicine Khon Kaen University Khon Kaen Thailand; 5 Department of Ophthalmology Faculty of Medicine Ramathibodi Hospital, Mahidol University Bangkok Thailand; 6 Department of Ophthalmology Rajavithi Hospital Bangkok Thailand; 7 Thai Red Cross Eye Bank Bangkok Thailand; 8 Department of Ophthalmology Faculty of Medicine Siriraj Hospital Mahidol University Bangkok Thailand; 9 Department of Ophthalmology Faculty of Medicine Chiang Mai University Chiang Mai Thailand; 10 Department of Ophthalmology Faculty of Medicine Songklanagarind Hospital, Prince of Songkla University Songkhla Thailand; 11 Department of Ophthalmology Mettapracharak Hospital Nakhon Pathom Thailand; 12 Department of Ophthalmology Phramongkutklao College of Medicine Bangkok Thailand; 13 Department of Ophthalmology Faculty of Medicine Thammasat University Pathum Thani Thailand

**Keywords:** blink rate, dry eye, smartphone application, maximum blink interval, prevalence, mHealth, epidemiology, screening, risk factors, symptoms, ophthalmology, vision

## Abstract

**Background:**

Dry eye (DE) is a chronic inflammatory disease of the ocular surface of the eye that affects millions of people throughout the world. Smartphone use as an effective health care tool has grown exponentially. The “Dry eye or not?” app was created to evaluate the prevalence of symptomatic DE, screen for its occurrence, and provide feedback to users with symptomatic DE throughout Thailand.

**Objective:**

The purpose of this study was to compare the prevalence of symptomatic dry eye (DE), blink rate, maximum blink interval (MBI), and best spectacle-corrected visual acuity (BSCVA) between people with and without symptomatic DE and to identify risk factors for symptomatic DE in Thailand.

**Methods:**

This cross-sectional study sourced data from the “Dry eye or not?” smartphone app between November 2019 and July 2020. This app collected demographic data, Ocular Surface Disease Index (OSDI) score, blink rate, MBI, BSCVA, and visual display terminal (VDT) use data. The criterion for symptomatic DE was OSDI score ≥13.

**Results:**

The prevalence of symptomatic DE among individuals using this smartphone app in Thailand was 85.8% (8131/9482), with the Northeastern region of Thailand having the highest prevalence, followed by the Northern region. Worse BSCVA (median 0.20, IQR 0.40; *P*=.02), increased blink rate (median 18, IQR 16; *P*<.001), reduced MBI (median 8.90, IQR 10.80; *P*<.001), female sex (adjusted OR 1.83; 95% CI 1.59-2.09; *P*<.001), more than 6 hours of VDT use (adjusted OR 1.59; 95% CI 1.15-2.19; *P*=.004), and lower than bachelor’s degree (adjusted OR 1.30; 95% CI 1.03-1.64; *P*=.02) were significantly associated with symptomatic DE. An age over 50 years (adjusted OR 0.77; 95% CI 0.60-0.99) was significantly less associated with symptomatic DE (*P*=.04).

**Conclusions:**

This smartphone DE app showed that the prevalence of symptomatic DE in Thailand was 85.8%. Signs and risk factors could be also evaluated with this smartphone DE app. Screening for DE by this app may allow for the development of strategic plans for health care systems in Thailand.

## Introduction

Dry eye (DE) is a chronic inflammatory disease with multifactorial etiology involving a loss of tear film homeostasis, leading to tear film instability and hyperosmolarity and triggering a vicious cycle of DE [1]. This condition affects millions of people globally with a prevalence in the range of 5%-30% in those over 50 years of age [2]. Previous research has identified many risk factors for DE, including older age, female sex, refractive surgery, connective tissue disease, low humidity environment, and use of visual display terminals (VDTs) [2]. Symptoms of dry eye are varied and include itching, burning, stinging, pain, photophobia, foreign body sensation, ocular redness, and blurred vision. Despite this, many people with DE symptoms remain unevaluated, undiagnosed, and untreated [3].

Smartphone use has grown globally at an exponential rate and has been proven as an effective health care tool for use by patients and physicians [4]. Many smartphone apps have been developed to support and empower patients, including apps for DE screening that evaluate lifestyle and associated risk factors [5-13]. We designed the “Dry eye or not?” app using Flutter by Google to identify individuals with a diagnosis of symptomatic DE, document DE symptoms, and assess blink rate, maximum blink interval (MBI) [14], best spectacle-corrected visual acuity (BSCVA), and risk factors associated with diagnosed symptomatic DE. This app was created by the Cornea and Refractive Surgery Society of Thailand. Because it was easy access and people in any region of the country could download this app, it was a convenient tool for evaluating the prevalence of symptomatic DE throughout the country and for screening and providing feedback to users with symptomatic DE, such as clinical advice.

The aim of this study was to estimate symptomatic DE prevalence and compare prevalence among regions of Thailand. In addition, this study aimed to compare blink rate, MBI, and BSCVA between individuals with and without symptomatic DE and to identify risk factors for this condition using “Dry eye or not?” app.

## Methods

### Study Participants

This cross-sectional study used the custom-designed “Dry eye or not?” smartphone app that was available for download in Thailand from November 2019 to July 2020. The app was released free of charge by the Cornea and Refractive Surgery Society of Thailand, a group of cornea and ocular surface disease experts, with no financial compensation. All voluntary users gave informed consent in electronic format. The inclusion criteria included individuals who were be able to read Thai language and had smartphones. Incomplete responses for blink rate, maximum blink interval, BSCVA, and the OSDI questionnaire were excluded.

### Ethics Approval

This study followed the tenets of the Declaration of Helsinki and was approved by the Institutional Review Board of the Faculty of Medicine, Chulalongkorn University, Thailand.

### Data Collection

**Figure 1:**
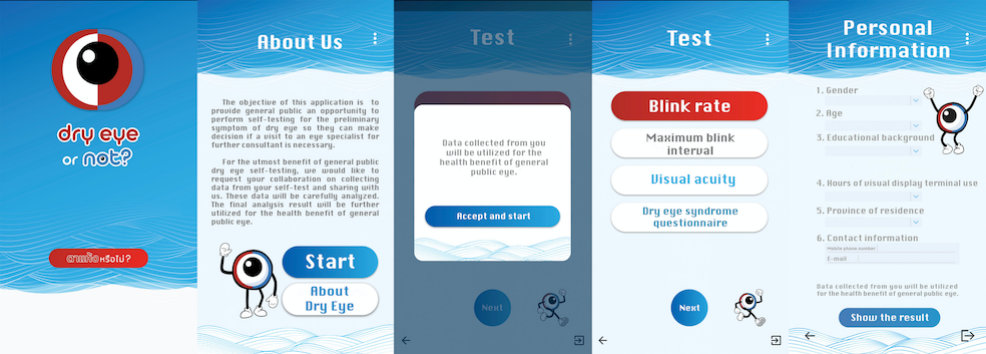
Screenshots of "Dry eye or not" app. Left to right: welcome screen, screen of information about this app, inform consent screen, measurement screen (blink rate, maximum blink interval, best spectacle-corrected visual acuity [BSCVA], Ocular Surface Disease Index [OSDI] questionnaire), and demographic characteristics of participants.

The app was freely downloadable via smartphone-based iOS and Android operating systems. This app collected data on blink rate (per minute), maximum blink interval [14] (seconds, secs), BSCVA (logMAR), DE symptoms, and demographic characteristics, as shown in Figure 1. The application programming interface (API) of blink detection in this app was used with a machine learning (ML) face detection kit developed by Google, which can recognize, locate, and determine the contours of facial features. We used this API to detect and record the number of eye blinks and MBI. Test instructions were displayed on smartphones before each test started, and the front camera was automatically accessed. Users were instructed to fit their face image to the camera display with a viewing distance of about 40 cm. Symptoms of DE were evaluated using the 12-item Ocular Surface Disease Index (OSDI) (Multimedia Appendix 1), with scores of ≥13 diagnostic of DE and severity classified as mild (13-22 points), moderate (23-32 points), and severe (33-100 points) [15]. Demographic data included age, sex, educational level (relative to bachelor’s degree), hours of VDT use per day, and region where each user was living.

#### Blink Rate and Maximum Blink Interval

Before commencing these tests, users were instructed to remove any spectacles and to blink normally and naturally. The blink test recorded the number of blinks per 30-second period and stored this as the number of blinks per minute. Before starting the MBI test, users were instructed to close their eyes in preparation and then to open them and keep them open for as long as possible. The time recording began when the eyes were opened, and the recorded duration was from this point to the first eye closure [14].

#### Best Spectacle-Corrected Visual Acuity

All participants were asked to wear their glasses before starting the test. The test began with tumbling E at a size equivalent to 20/40 Snellen. If the participant failed to select the correct answer, chose to skip the question, or did not provide any answer within 2 seconds, the tumbling E size was increased by 1 Snellen line. However, if the participant answered correctly on 2 consecutive occasions, the tumbling E became smaller by 1 Snellen line.

### Statistical Analysis

Demographics and baseline clinical characteristics were analyzed in frequency and percentage. The categorical data were compared using Pearson chi-square test. Blink rate, MBI, and BSCVA (logMAR) were compared between individuals with and without symptomatic DE using a Wilcoxon rank-sum test and presented as median with interquartile range (IQR). Moreover, an unpaired Student *t* test was also used to compared OSDI score between groups and presented as mean with standard deviation. Kruskal-Wallis with Dunn test for multiple comparison was used to compare the BSCVA between individuals with different severity levels of symptomatic DE and without symptomatic DE. Univariate and multivariate logistic regressions, presented in crude and adjusted odds ratio (OR) with 95% CI, were used to assess the associations between risk factors and symptomatic DE. For all analyses, a *P* value of .05 was the criterion for statistical significance, and Stata software version 15.1 (StataCorp) was used.

## Results

### Initial Findings

A total of 13,228 individuals used the app. All were volunteers aged above 15 years of age and were Thai citizens living in Thailand. However, data from 3746 users were incomplete and were thus excluded from analysis. The complete data of the excluded participants included demographic and baseline characteristics and OSDI scores. A sensitivity analysis of complete data between included and excluded participants was done and is shown in Multimedia Appendix 2. There was a statistically significant difference in age factor (*P*<.001) and OSDI scores (*P*<.001). However, the percentage of participants in each age group was similarly distributed, and the OSDI scores between the included group (mean 30.59, SD 17.94) and the excluded group (mean 33.68, SD 19.15) was not clinically significantly different. As a result, data from 9482 users were analyzed. Of these, 1811 (19.1%) were men and 7671 (80.9%) were women. The baseline characteristics of the participants, including age, VDT use per day, educational level, and regions of residence including Northern, Northeastern, Eastern, Western, Central, Southern, and capital city (Bangkok), are shown in Table 1. A comparison of these characteristics between individuals with and without symptomatic DE is presented in Table 2.

**Table 1:** Demographic and baseline characteristics of participants (N=9482).

Characteristics		Values, n (%)
**Age (years)**		
	15-20	1883 (19.9)
	21-30	4612 (48.6)
	31-40	1342 (14.2)
	41-50	803 (8.5)
	>50	842 (8.9)
**VDT^a^ use per day**		
	Less than 1 hour	278 (2.9)
	1-4 hours	985 (10.4)
	>4-6 hours	2432 (25.7)
	>6-8 hours	2754 (29)
	>8 hours	3033 (32)
**Educational level**		
	Lower than bachelor’s degree	1831 (19.3)
	Bachelor’s degree	6281 (66.3)
	Higher than bachelor’s degree	1370 (14.5)
**Regions of participants’ residence**		
	Northern region	727 (7.8)
	Northeastern region	934 (10)
	Central region excluding Bangkok city	1,975 (21.1)
	Bangkok city	4316 (46.1)
	Eastern region	656 (7)
	Western region	196 (2.1)
	Southern region	561 (6)

^a^VDT: visual display terminal.

**Table 2:** Comparison of characteristics of participants with and without symptomatic dry eye.

Characteristics			Symptomatic dry eye (n, %)					*P* value
			Without		With			
**Age (years)**								<.001
	15-20	231 (17.1)		1652 (20.3)				
	21-30	566 (41.9)		4046 (49.8)				
	31-40	230 (17)		1112 (13.7)				
	41-50	134 (9.9)		669 (8.2)				
	>50	190 (14.1)		652 (8)				
**Sex**								<.001
	Female	945 (69.9)		6726 (82.7)				
	Male	406 (30.1)		1405 (17.3)				
**VDT^a^ use (hours/day)**								<.001
	Less than 1 hour	61 (4.5)		217 (2.7)				
	1-4 hours	199 (14.7)		786 (9.7)				
	>4-6 hours	392 (29)		2040 (25.1)				
	>6-8 hours	360 (26.7)		2694 (29.4)				
	>8 hours	339 (25.1)		2694 (33.1)				
**Educational level**								<.001
	Lower than bachelor’s degree	237 (17.5)		1594 (19.6)				
	Bachelor’s degree	851 (63)		5430 (66.8)				
	Higher than bachelor’s degree	263 (19.5)		1107 (13.6)				

^a^VDT: visual display terminal.

### Prevalence of Symptomatic Dry Eye

Of the 9482 participants, 8131 (85.8%) were diagnosed with symptomatic DE. The prevalence differed significantly between regions (*P*<.001), as shown in Table 3. In addition, the prevalence of subgroups of symptomatic DE (normal, mild, moderate, and severe grade) were also significantly different among regions (*P*<.001), as shown in Table 4.

**Table 3:** Prevalence of symptomatic dry eye in each region of Thailand.

Region		Symptomatic dry eye (n, %)	
	Without, n=1334 (14.2)		With, n=8031 (85.8)
Northern	82 (6.1)		645 (8.0)
Northeast	96 (7.2)		838 (10.4)
Central (except Bangkok)	272 (20.4)		1703 (21.2)
Bangkok	683 (51.2)		3633 (45.3)
Eastern	85 (6.4)		571 (7.1)
Western	33 (2.5)		163 (2)
Southern	83 (6.2)		478 (6)

**Table 4:** Prevalence of symptomatic dry eye subgroups in each region of Thailand.

Region	Symptomatic dry eye, n (%)			
	Normal, n=1334 (14.2)	Mild, n=2183 (23.3)	Moderate, n=2024 (21.6)	Severe, n=3824, (40.8)
Northern	82 (11.3)	162 (22.3)	159 (21.9)	324 (44.5)
Northeast	96 (10.3)	186 (19.9)	198 (21.2)	454 (48.6)
Central (except Bangkok)	272 (13.8)	484 (24.5)	426 (21.6)	793 (40.1)
Bangkok	683 (15.8)	1,030 (23.9)	938 (21.7)	1,665 (38.6)
Eastern	85 (12.9)	143 (21.8)	144 (22)	284 (43.3)
Western	33 (16.8)	47 (24)	37 (18.9)	79 (40.3)
Southern	83 (14.8)	131 (23.3)	122 (21.8)	225 (40.1)

### Blink Rate Per Minute and Maximum Blink Interval

Blink rate differed significantly between participants with (median 18, IQR 16 blinks) and without (median 16, IQR 16) blinks) symptomatic DE (*P*<.001). A significant difference was also found in MBI between participants with and without symptomatic DE (median 8.90, IQR 10.80 seconds vs median 8.90, IQR 14.8 seconds, respectively; *P*<.001). Binary logistic regression showed a significant association between the DE group and blink rate (univariate OR 1.02; 95% CI 1.02-1.03; *P*<.001) and between the DE group and MBI (univariate OR 0.98; 95% CI 0.98-0.99; *P*<.001). After controlling for risk factors, including age, sex, VDT use, and educational level, this association was sustained in both blink rate (multivariate-adjusted OR 1.01; 95% CI 1.01-1.02; *P*<.001) and MBI (multivariate-adjusted OR 0.98; 95% CI 0.98-0.99; *P*<.001).

### Best Spectacle-Corrected Visual Acuity

The BSCVA (logMAR) in users without symptomatic DE (median 0.20, IQR 0.40; mean 0.22, SD 0.21) was significantly different from that of the symptomatic DE group (median 0.20, IQR 0.40; mean0.23, SD 0.21; *P*=.02). Mean BSCVA was 0.22 (95% CI 0.21-0.23) in users without symptomatic DE; among symptomatic users, the mean was 0.22 (95% CI 0.21-0.23) in mild cases, 0.22 (95% CI 0.21-0.23) in moderate cases, and 0.24 (95% CI 0.24-0.25) in severe cases. Moreover, the median BSCVA in users without symptomatic DE and with mild, moderate, and severe symptomatic DE was 0.2 (IQR 0.4), and there was a statistically significant difference (*P*<.001). Pairwise comparison revealed a difference between the severe group and each of the three other subgroups, normal (*P*<.001), mild (*P*<.001), and moderate (*P*<.001), but not between the latter three groups (normal vs mild, *P*=.17; normal vs moderate, *P*=.26; mild vs moderate; *P*=.36).

### Risk Factors

As shown in Table 5, binary logistic regression found that symptomatic DE was more prevalent in female users (multivariate-adjusted OR 1.83; 95% CI 1.59-2.09; *P*<.001), those reporting VDT use of >6-8 hours per day (multivariate-adjusted OR 1.59; 95% CI 1.15-2.19; *P*=.005), and those with an educational level lower than bachelor’s degree (multivariate-adjusted OR 1.30; 95% CI 1.03-1.64; *P*=.02). OSDI scores were significantly higher in female (mean 31.55, SD 17.87) than male (mean 26.53, SD 17.67, *P*<.001) users, in those with6 hours (mean 32.50, SD 18.27) than those with 6 hours of VDT use (mean 27.59, SD 16.98, *P*<.001), and in those with an educational level lower than bachelor’s degree and bachelor’s degree (mean 31.23, SD 17.94, *P*<.001) versus an educational level higher than bachelor’s degree (mean 26.81, SD 17.47). Moreover, the Southern, Western, and Central regions and Bangkok had significantly less impact on symptomatic DE compared with the Northeastern region in binary logistic regression.

**Table 5:** Risk factors for symptomatic dry eye compared with those without dry eye.

			Univariate OR^a^ (95% CI)		*P* value		Multivariate-adjusted OR (95% CI)		*P* value
**Age (years)**									
	15-20	1 [reference]		N/A^b^		1 [reference]		N/A	
	21-30	0.99 (0.85, 1.18)		.97		1.07 (0.88, 1.29)		.48	
	31-40	0.68 (0.55, 0.82)		<.001		0.85 (0.67, 1.06)		.15	
	41-50	0.69 (0.55, 0.88)		.002		0.99 (0.76, 1.28)		.94	
	>50	0.47 (0.38, 0.58)		<.001		0.77 (0.60, 0.99)		.04	
	Sex (female vs male)	2.06 (1.81, 2.34)		<.001		1.83 (1.59, 2.09)		<.001	
**VDT^c^ use (hours/day)**									
	Less than 1 hour	1 [reference]		N/A		1 [reference]		N/A	
	1-4 hours	1.11 (0.80, 1.53)		.53		1.08 (0.77, 1.50)		.67	
	>4-6 hours	1.49 (1.10, 2.02)		.01		1.31 (0.96, 1.80)		.09	
	>6-8 hours	1.87 (1.39, 2.55)		<.001		1.59 (1.15, 2.19)		.005	
	>8 hours	2.29 (1.69, 3.12)		<.001		1.86 (1.35, 2.58)		<.001	
**Educational level**									
	Higher than bachelor’s degree	1 [reference]		N/A		1 [reference]		N/A	
	Lower than bachelor’s degree	1.58 (1.31, 1.92)		<.001		1.30 (1.03, 1.64)		.02	
	Bachelor’s degree	1..51 (1.30, 1.76)		<.001		1.18 (0.99, 1.39)		.06	
**Region**									
	Northeast	1 [reference]		N/A		1 [reference]		N/A	
	Northern	0.90 (0.67, 1.23)		.51		0.93 (0.68, 1.27)		.64	
	Central (except Bangkok)	0.72 (0.56, 0.92)		.008		0.76 (0.59, 0.98)		.03	
	Bangkok	0.61 (0.49, 0.76)		<.001		0.64 (0.51, 0.81)		<.001	
	Eastern	0.77 (0.56, 1.05)		.10		0.79 (0.57, 1.08)		.13	
	Western	0.57 (0.37, 0.87)		.009		0.62 (0.40, 0.97)		.03	
	Southern	0.66 (0.48, 0.90)		.01		0.68 (0.49, 0.93)		.02	

^a^OR: odds ratio

^b^N/A: not applicable.

^c^VDT: visual display terminal.

## Discussion

### Principal Results

Technology has evolved rapidly in recent years, and smartphones provide one example of this, having transformed dramatically to provide sophisticated communication and data access, including health information [16]. In this study, we used smartphone technology by creating the app “Dry eye or not?” to evaluate the countrywide and regional prevalence of symptomatic DE in Thailand (85.8%). This app enables recording of blink rate (median 18, IQR 16 blinks) and maximum blink interval (median 8.90, IQR 10.80 seconds) associated with DE [14]. Moreover, we used this app to collect BSCVA and demographic data including age, sex, hours of VDT use per day, regions where individuals lived, and educational levels to assess users’ relationship with diagnosed symptomatic DE.

The prevalence of symptomatic DE in Thailand in our study was 85.8%, higher than the 34% prevalence reported in 2006 [17] and 14.2% in 2012 [18]. It was also much higher than the 5% to 50% prevalence reported by the Tear Film and Ocular Surface Society’s Dry Eye Workshop Study II in 2017 [19], which included prevalence of either or both DE symptoms and signs. However, the symptomatic DE prevalence reported by Inomata et al [6] in 2019 based on data collected using a smartphone app was 74% in Japan. These prevalence values indicate an increasing trend over time [20]. Moreover, Asian race is a known risk factor for DE; consistent with this, prevalence in this study and others including Asian populations is higher than in studies including other races [6,20]. In addition, the fact that both this study and Inomata et al [20] used smartphone apps for data collection may have biased results toward users with high daily VDT use, another risk factor for DE. In this study, the prevalence of symptomatic DE was highest in the Northeastern region (89.7%), followed by the Northern region (88.7%) of Thailand. The prevalence of severe symptomatic DE was higher than other severity grades in every region of Thailand, and the highest prevalence of severe DE was also in the Northeastern region (48.6%), followed by the Northern region (44.5%). Moreover, binary logistic regression analysis showed that the Northeastern region had a significantly greater impact on symptomatic DE compared with Southern, Western, and Central regions and Bangkok. According to climatic data from the Thai Meteorological Department, the Northern, Northeastern, and Central regions have lower relative humidity than other regions, but the annual average temperature is similar among the regions. High temperature, low humidity, and wind are known risk factors for DE [19,21]. Additionally, air pollutants including ozone (O_3_), particulate matter 2.5 (PM 2.5), and sulfur dioxide (SO_2_) have been identified as risk factors for DE [22]. Relatively high concentrations of O_3_ are found in Central, Northeastern, and Northern regions, high SO_2_ concentrations in Central and Northeastern regions [23], and high PM 2.5 concentrations in Bangkok, Central, and Northern regions [24]. High pollution and low humidity may explain the high prevalence of symptomatic DE in Northern and Northeastern regions of Thailand in this study. The Central region also has high pollution and low relative humidity but had a lower prevalence of symptomatic DE in this study. One possible explanation for this is that the population in urban locations in the Central region may be equipped with better health care education, knowledge of the health care system, and access to medication.

Blinking is well established as an associated factor in ocular surface sensation and is commonly quantified by measuring the blink rate or its reciprocal value, such as MBI [14]. This relationship has been demonstrated by many studies that show a link between an increased blink rate in DE and ocular surface irritation, surface dryness, or an unstable tear film, suggesting a blink rate test as a screening tool for DE [25-28]. The rate of spontaneous eye blinking has a complex relationship with ocular surface health, including DE status [28]. In this study, the median spontaneous blink rate (18 blinks per minute) in users with symptomatic DE was significantly higher than in the normal group. The mean blink rate of DE groups in previous studies ranged from 28.55 blinks per minute to 15.32 blinks per 20 seconds and varied considerably, including in this study [25-28]. Interblink interval (IBI) and MBI have both been shown by many studies to be related to DE, with a mean IBI in DE ranging from 2.56 seconds to 12.52 seconds, while the criterion MBI was reported as 12.4 seconds with a sensitivity of 82.5% and a specificity of 51% [14,28,29]. Similarly, the mean MBI in our study was 11.80 seconds with a median of 8.9 seconds. Moreover, according to binary logistic regression, increased blink rate and decreased MBI were associated with symptomatic DE. These findings suggest that blink rate and MBI measured using smartphone technology may be used as screening tools for symptomatic DE, promoting self-diagnosis of symptomatic DE. Because research suggests disagreement between signs and symptoms of DE [30], the efficacy and accuracy of the app developed in this study may be further improved by incorporating factors relating to both signs and symptoms of DE.

DE is a disease associated with ocular surface inflammation, which causes irregularity of the ocular surface and reduced uncorrected visual acuity, the latter having been demonstrated by several studies in patients with DE. Moreover, BSCVA could be reduced in a severe grade of DE. In 2019, Zczotka-Flynn^ ^et al [31] also reported that BSCVA was reduced in individuals with worse mean OSDI score [32]. In this study, the BSCVA in the normal group was statistically significantly better than in the symptomatic DE group, and a severe grade of symptomatic DE group had statistically significantly worse BSCVA compared with other groups from the subgroup analysis. The symptomatic DE groups were believed to have instability of tear film layer, which resulted in the irregularity of ocular surface and consequently had a negative impact on optical quality as the air-tear film interface contributes the most to the ocular refractive power [33].

In this study, most participants (6495/9482, 66%) were under 30 years old. The small proportion of older participants in this study is similar to previous research using a smartphone app [6]. Previous studies have shown that the prevalence of DE increases with age [19,20]. However, in this study, participants aged over 50 were less likely to have symptomatic DE than those under 30 years of age. This finding is in accordance with some previous studies [6,34] indicating that older participants may be less likely to report ocular symptoms than younger individuals due to reduced corneal sensitivity in older participants resulting from reductions in corneal density and substance P (a neuropeptide secreted by sensory nerves that modulates nociceptive pain) in older age [35]. Moreover, the proportion of older participants in this study was low, and this relatively small sample of older individuals may have affected prevalence estimates. In addition, younger people tend to report more daily hours of VDT use, and this may have contributed to DE in younger participants.

The female sex has been identified and widely reported as an important factor in DE [19] and was also found to be a risk factor in this study. Sex hormones have roles in tear synthesis. Androgen binding to receptors on the meibomian glands leads to increased lipid synthesis and secretion, while estrogen (predominantly in females) binding lessens lipid production [36].

VDT use has been identified as a risk factor for DE in many studies, but the number of hours of VDT use per day in individuals with DE varies between studies from more than 4 to more than 8 hours a day [6,19,37-39]. In this study, VDT use for more than 6 hours a day was significantly associated with symptomatic DE. The difference in reported periods of VDT use constituting a risk factor for DE may be due to the different criteria for DE diagnosis and different ethnicities included. At present, smartphone and other electronic device displays are increasingly used worldwide, and people, particularly those who are young, spend more time on these devices than was the case in the past. These factors could lead to an increasing prevalence of DE in the future.

According to the logistic regression analysis, an educational level lower than a bachelor’s degree was found to be a significant factor for symptomatic DE in this study. Thus, the percentage of symptomatic DE participants was highest in the lowest educational level group. After further investigation, we found that a lower educational level corresponds to a younger age group. The majority of people with a bachelor’s degree or lower had VDT>8 hours per day. The potential reason for the association between lower education and symptomatic DE was that most participants with a lower level of education were aged 15-30 years old, and they had the highest proportion of having 8 hours or more of VDT use. Therefore, this could signify a generational difference, in that a younger age group was associated with a greater VDT use.

### Limitations

We acknowledge some limitations in this study, which were similar to those found in other web- or app-based studies. First, our study defined DE diagnosis solely based on the OSDI questionnaire, representing only symptoms of DE. Second, our participants included only those with smart phone capability, thereby restricting our group to younger participants with a relatively high socioeconomic status and education level [12]. Older participants might have a limited ability to use smartphone because of their physical limitations. Third, symptomatic DE individuals were more likely to participate in our project since their interest in alleviating DE may have acted as a motivating factor, whereas those with no DE symptoms may ignore this app [3]. However, this potential problem was alleviated by the large sample size in this study, which recruited participants from diverse geographic regions throughout Thailand. No clinical examination was possible in this remote data collection format; as a result, we tested blink rate and MBI using the smartphone to assess their association with symptomatic DE. Another limitation of this study has to do with the reliability of the blink rate and MBI measurements, since they were carried out in different temperature and humidity conditions; despite this limitation, this study demonstrated the feasibility of blink rate measurement using a smartphone app and showed the link between blink rate and symptomatic DE. Future research incorporating such tests conducted using this app and conventional clinical examination will help improve and validate this convenient screening tool for DE diagnosis. The last limitation was that at the time of this study, there was no published study that validated the OSDI questionnaires in the Thai language; however, that study is currently underway.

### Conclusions

According to the results of this crowdsourced study, in which the prevalence of symptomatic DE in Thailand was 85.8%, blink rate, MBI, BSCVA, and risk factors for DE may be evaluated using a smartphone app. Moreover, blink rate and MBI recorded in this way may identify people at risk of symptomatic DE. The Northeastern region of Thailand showed the highest prevalence of symptomatic DE, followed by the Northern region. Increased blink rate, reduced MBI, and reduced BSCVA were associated with symptomatic DE. Younger age was more strongly associated with symptomatic DE than older age. Female sex, more than 6 hours daily VDT use, and a lower education level were also significant risk factors of symptomatic DE. These findings will lead to further research on the use of smartphone app screening tools with high sensitivity and specificity for diagnosis of DE, enabling early diagnosis and treatment of this condition. This approach to screening for DE may aid the development of strategic plans for health care systems in Thailand.

## Appendix

Multimedia Appendix 1 Ocular Surface Disease Index questionnaire.

Multimedia Appendix 2 Sensitivity analysis.

## Abbreviations

API: application programming interface
BSCVA: best spectacle-corrected visual acuity
DE: dry eye
IBI: interblink interval
IQR: interquartile range
MBI: maximum blink interval
ML: machine learning
O_3_: ozone
OSDI: Ocular Surface Disease Index
PM 2.5: particulate matter 2.5
SO_2_: sulfur dioxide
VDT: visual display terminal
