# Supplementary material for: Assessing the Risk Factors For Diagnosed Symptomatic Dry Eye Using a Smartphone App: Cross-sectional Study
Source: JMIR Mhealth Uhealth. 2022 Jun 22;10(6):e31011. doi: 10.2196/31011 (PMC9260529; doi:10.2196/31011)
Supplement: Multimedia Appendix 1 [file mhealth_v10i6e31011_app1.docx]

**Multimedia Index 1. Dry eye syndrome questionnaires (Ocular Surface Disease Index)[15]**

|  | All of the time | Most of the time | Half of the time | Some of the time | None of the time |
| --- | --- | --- | --- | --- | --- |
| Have you experienced any of the following during the last week | | | | | |
| 1.Eyes that are sensitive to light? |  |  |  |  |  |
| 2.Eyes that feel gritty? |  |  |  |  |  |
| 3.Painful or sore eyes? |  |  |  |  |  |
| 4.Blurred vision? |  |  |  |  |  |
| 5.Poor vision? |  |  |  |  |  |
| Have problems with your eyes limited you in performing any of the following during the last week | | | | | |
| 6.Reading? |  |  |  |  |  |
| 7.Driving at night? |  |  |  |  |  |
| 8.Working with a computer or bank machine (ATM)? |  |  |  |  |  |
| 9.Watching TV? |  |  |  |  |  |
| Have your eyes felt uncomfortable in any of the following situations during the last week | | | | | |
| 10.Windy conditions? |  |  |  |  |  |
| 11.Places or areas with low humidity (very dry)? |  |  |  |  |  |
| 12.Areas that are air conditioned? |  |  |  |  |  |
