# Supplementary material for: Assessing the Risk Factors For Diagnosed Symptomatic Dry Eye Using a Smartphone App: Cross-sectional Study
Source: JMIR Mhealth Uhealth. 2022 Jun 22;10(6):e31011. doi: 10.2196/31011 (PMC9260529; doi:10.2196/31011)
Supplement: Multimedia Appendix 2 [file mhealth_v10i6e31011_app2.docx]

**Multimedia index 2. Sensitivity analysis between included and excluded data**

| **Demographic and baseline characteristics** | **Included**  **(n=9,482)** | **Excluded**  **(n=3,746)** | ***P* values** |
| --- | --- | --- | --- |
| **Age years, number(%)** |  | | |
| 15-20 | 1,883 (19.9%) | 695 (18.6%) | <.001 |
| 21-30 | 4,612 (48.6%) | 1,852 (49.4%) |  |
| 31-40 | 1,342 (14.2%) | 469 (12.5%) |  |
| 41-50 | 803 (8.5%) | 311 (8.3%) |  |
| >50 | 842 (8.9%) | 419 (11.2%) |  |
| **Sex, number (%)** |  | | |
| Female | 7,671 (80.9%) | 3,000 (80.1%) | .81 |
| **Visual display terminal use (hours/day)** |  | | |
| Less than 1 hour | 278 (2.9%) | 89 (2.4%) | .16 |
| 1-4 hours | 985 (10.4%) | 380 (10.1%) |  |
| >4-6 hours | 2,432 (25.7%) | 974 (26.0%) |  |
| >6-8 hours | 2,754 (29.0%) | 1,147 (30.6%) |  |
| >8 hours | 3,033 (32.0%) | 1,156 (30.9%) |  |
| **Educational level, number (%)** |  | | |
| Lower than Bachelor’s degree | 1,831 (19.3%) | 705 (18.8%) | .81 |
| Bachelor’s degree | 6,281 (66.2%) | 2,498 (66.7%) |  |
| Higher than Bachelor’s degree | 1,370 (14.5%) | 543 (14.5%) |  |
| **OSDI scores, mean (SD)** | 30.59 (17.94) | 33.68 (19.15) | <0.001 |
